# Structure activity relationship studies on Amb639752: toward the identification of a common pharmacophoric structure for DGKα inhibitors

**DOI:** 10.1080/14756366.2019.1684911

**Published:** 2019-11-05

**Authors:** Suresh Velnati, Alberto Massarotti, Annamaria Antona, Maria Talmon, Luigia Grazia Fresu, Alessandra Silvia Galetto, Daniela Capello, Alessandra Bertoni, Valentina Mercalli, Andrea Graziani, Gian Cesare Tron, Gianluca Baldanzi

**Affiliations:** a Department of Translational Medicine, University of Piemonte Orientale, Novara, Italy; b Institute for Research and Cure of Autoimmune Diseases, CAAD, University of Piemonte Orientale, Novara, Italy; c Department of Pharmaceutical Sciences, University of Piemonte Orientale, Novara, Italy; d Department of Health Sciences, School of Medicine, University of Piemonte Orientale, Novara, Italy; e Palliative Care Division, A.S.L., Vercelli, Italy; f Università Vita-Salute San Raffaele, Milan, Italy; g Department of Molecular Biotechnology and Health Sciences, Molecular Biotechnology Center, University of Torino, Turin, Italy

**Keywords:** Diacylglycerol kinase, kinase inhibitors, structure–activity relationship, enzyme assays, molecular modelling

## Abstract

A series of analogues of Amb639752, a novel diacylglycerol kinase (DGK) inhibitor recently discovered by us via virtual screening, have been tested. The compounds were evaluated as DGK inhibitors on α, θ, and ζ isoforms, and as antagonists on serotonin receptors. From these assays emerged two novel compounds, namely **11** and **20**, which with an IC_50_ respectively of 1.6 and 1.8 µM are the most potent inhibitors of DGKα discovered to date. Both compounds demonstrated the ability to restore apoptosis in a cellular model of X-linked lymphoproliferative disease as well as the capacity to reduce the migration of cancer cells, suggesting their potential utility in preventing metastasis. Finally, relying on experimental biological data, molecular modelling studies allow us to set a three-point pharmacophore model for DGK inhibitors.

## 1. Introduction

Diacylglycerol kinases (DGKs) are a large family of enzymes that share a common catalytic activity: the phosphorylation of diacylglycerol (DAG) to phosphatidic acid (PA). Remarkably, both the substrate (DAG) and the product (PA) of the DGK-catalysed reaction, are bioactive lipids that can act as second messengers^1^. DGK activity consequently serves as a switch to simultaneously dampen DAG-mediated signals and boost PA-mediated signals^2^. Ten mammalian DGK isoforms (α, β, γ, δ, ε, ζ, η, θ, ι, and к) have been identified and divided into five groups (type I–V) according to their structural features^3^^,^^4^. The expression of these isoforms varies depending on the cell type. Among the 10 isoforms, the α isoform is among the most studied and characterised. This kinase is highly expressed in the brain, spleen, and thymus and, along with θ isoform, in the bone marrow. This enzyme is also highly expressed in T-lymphocytes, where it acts together with DGKζ as negative regulator of the T-cell receptor (TCR) response, and a mediator of IL-2 mediated proliferation^3^^,^^5^. The biological relevance of DGKα is best demonstrated in patients with X-linked lymphoproliferative disease (XLP-1), who experience life-threatening, uncontrolled accumulation of CD8^+^ T cells in response to the Epstein–Barr virus (EBV) infection^6^. In those patients, germline mutations of the adaptor protein SAP (SH2D1A) perturb TCR signalling and render DGKα constitutively active^7^. Deregulated DGKα activity renders patient-derived lymphocytes resistant to reactivation-induced cell death (RICD). Thus, antigen-activated lymphocytes accumulate in lymphonodes and liver, resulting in severe immunopathology^8^. Importantly, DGKα inhibitors restore RICD sensitivity *in vitro* and *in vivo*, thus avoiding immunopathology and suggesting a putative therapeutic use of those molecules in XLP-1^9^.

Apart from T-cell regulation, DGKα also plays a role in cancer, mediating numerous aspects of cancer cell progression including survival^10^^,^^11^, migration and invasion of cancer cells^12–14^. In particular, it has been reported that DGKα is over expressed in hepatocellular carcinoma^15^, and melanoma cells^11^ while other reports suggested that the growth of colon and breast cancer cell lines was significantly inhibited by DGKα-siRNA^16^ and DGKα/atypical PKC/β1 integrin signalling pathway was crucial for matrix invasion of breast carcinoma cells^17^. In addition, expression is also higher in lymphonodal metastasis than in breast and gastric original tumour^18^^,^^19^. Finally, knock down of DGKα impairs glioblastoma tumorigenesis^20^^,^^21^.

**Figure 1.**
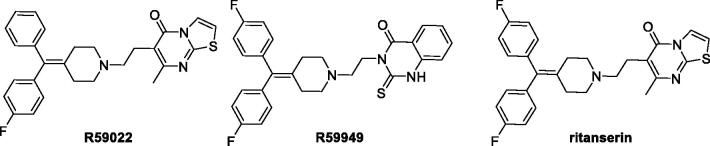
Three of the most studied DGKα inhibitors.

For all these reasons, the identification of strong and selective DGKα inhibitors, it is an important field of research. To date, only a handful of two-digit micromolar inhibitors of DGKα have been identified, but only three were the most characterised, namely, R59949, R59022, and ritanserin (Figure 1).

In our assay system, R59949 and R59022 have an IC_50_ of 11 and 20 µM, respectively^9^. Their efficacy has been evaluated *in vivo* studies on mice, and is limited by their rapid clearance (*t*_1/2_=∼2 h)^22^. Furthermore, these two inhibitors are also able to target different isoforms of DGK, in particular R59022 acts on type III and V (ε e θ), while R59949 on type I and II (γ, δ e κ)^23^^,^^24^ and a study conducted by Boroda et al. recently demonstrated their strong antagonistic activity on 5-HT_2_ receptors (R59022 IC_50_ 5HT_2A_=2.2 nM; R5994 IC_50_ 5HT_2A_=9.2 nM)^25^.

A search on ChEMBL database^26^ (https://www.ebi.ac.uk/chembl/) show how these two molecules have activity at the same range of concentration with other biological targets, behaving like a sort of promiscuous ligands. Ritanserin, a well-known serotoninergic antagonist, is structurally similar to R59022, differing for an H-F isosteric substitution on a phenyl ring. Despite this small modification, Boroda et al. showed that ritanserin was a DGKα inhibitor (IC_50_=15 µM) more potent than R59022 and R59949 and with a better pharmacokinetic profile (*t*_1/2_=40 h in human)^25^. However, the comparison of ritanserin IC_50_ as serotonin antagonist and as DGKα inhibitor, 0.9 nM and 15,000 nM, respectively, reveal that ritanserin is much a powerful serotonin antagonist than a DGK inhibitor. In addition, ritanserin is also a potent inhibitor on dopaminergic receptors with an IC_50_ of 69 nM^27^.

**Figure 2.**
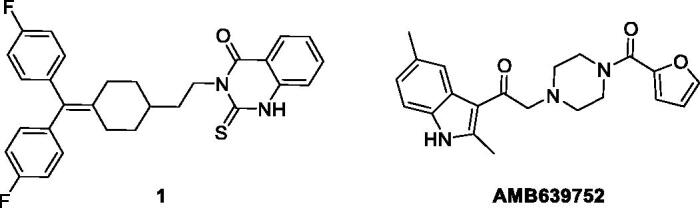
Structures of the deaza analogue of R59949 and Amb639752.

Due to these drawbacks, at the beginning, in order to eliminate the strong serotoninergic activity of R59949, we reasoned to replace its protonable nitrogen atom, which at physiologically pH mimics the amino group of serotonin, with a carbon atom. We decided therefore to synthesise compound **1** (Figure 2) (see supporting information for its synthesis and a complete characterisation) and to test it as DGKα inhibitor.

Interestingly, the compound was totally devoid of inhibitory activity on the enzyme, showing the importance of the basic nitrogen atom not only for the anti-serotoninergic activity, but also for the interaction with the kinase. With this in mind, we recently used an in-silico approach based on chemical homology with the two commercially available DGKα inhibitors R59022 and R59949 using the programmes ROCS^28^ and EON^29^. From this study, we identified a compound, Amb639752 (Figure 2), featuring a lower IC_50_ for DGKα than ritanserin (IC_50_=17 µM), a better selectivity for the α-isoform and devoid of anti-serotoninergic activity. Along with CU-3, which features an IC_50_ of 0.6 µM on DGKα^30^ but contains a reactive Michael acceptor^31^, Amb639752 is the most effective pharmacological tool available to study DGKα^9^. In this manuscript, we report the structure–activity studies on Amb639752 and, in combination with data on ritanserin, the generation of a pharmacophore model for this class of compounds, which could be useful for the identification of other potential DGKα inhibitors.

## 2. Methods

### 2.1. Chemistry procedures

Commercially available reagents and solvents were used without further purification. Toluene were distilled immediately before use from Na/benzophenone under a slight positive atmosphere of dry nitrogen. Dichloromethane was dried by distillation from P_2_O_5_ and stored on activated molecular sieves (4 Å). When needed the reactions were performed in flame- or oven-dried glassware under a positive pressure of dry nitrogen. Melting points were determined in open glass capillary with a Stuart scientific SMP3 apparatus and are uncorrected. All compounds were checked by IR (FT-IR THERMO-NICOLET AVATAR), ^1^H and 13C APT (JEOL ECP 300 MHz spectrometer), and mass spectrometry (Thermo Finningan LCQ-deca XP-plus, San Jose, CA) equipped with an ESI source and an ion trap detector. Chemical shifts are reported in parts per million (ppm). Flash column chromatography was performed on silica gel (Merck Kieselgel 60, 230–400 mesh ASTM, Kenilworth, NJ). Thin layer chromatography (TLC) was carried out on 5 × 20 cm plates with a layer thickness of 0.25 mm (Merck Silica gel 60 F_254_, Kenilworth, NJ). When necessary they were developed with KMnO_4_ reagent. Purity of tested compounds was established by elemental analysis. Elemental analysis (C, H, N) of the target compounds is within ±0.4% of the calculated values, confirming ≥95% purity.

#### 2.1.1. Preparation of 2-chloro-1-(2,6-dimethyl-1H-indol-3-yl)ethan-1-one (5)

In a Schlenk tube, under nitrogen, 2,6-dimethyl-1*H*-indole (**3**) (0.20 g, 1.38 mmol, 1 eq) was dissolved in 4 mL of dichloroethane dry and 0.25 mL of DBU (1.66 mmol, 1.2 eq) were added. The resulting solution was heated at 90 °C. When reached this temperature, chloroacetyl chloride (**4**) (0.12 mL, 1.52 mmol, 1.1 eq) was added. The reaction was stirred for 30 min, then solvent was evaporated and the crude purified by column chromatography using PE/EtOAc 7:3 and PE/EtOAc 5:5 as eluants to give 270 mg of product as violet solid: yield 90%; m.p. 243.7–244.2 °C; ^1^H NMR (300 MHz, DMSO-d_6_) *δ* 11.88 (br s, NH), 7.85 (d, *J* = 7.9 Hz, 1H), 7.18 (s, 1H), 6.99 (d, *J* = 8.0 Hz, 1H), 4.89 (s, 2H), 2.68 (s, 3H), 2.39 (s, 3H). MS (ESI) *m/z*: 222 [M + H]^+^.

#### 2.1.2. Preparation of tert-butyl 4-(2-(2,6-dimethyl-1H-indol-3-yl)-2-oxoethyl)piperazine-1-carboxylate (6)

Under nitrogen, 200 mg of **5** (0.90 mmol, 1 eq) was dissolved in toluene dry, then *N*-Boc-piperazine (0.17 g, 0.90 mmol, 1 eq), K_2_CO_3_ (0.32 g, 2.25 mmol, 2.5 eq), and KI (0.015 g, 0.09 mmol, 0.1 eq) were added. The reaction was heated at 80 °C overnight. Solvent was evaporated and the crude was purified by column chromatography using PE/EtOAc 4:6 and PE/EtOAc 2:8 as eluants to give 215 mg of product as yellow amorphous solid: yield 63%; ^1^H NMR (300 MHz, CDCl_3_) *δ* 8.52 (br s, NH), 7.77 (d, *J* = 8.2 Hz, 1H), 7.12 (s, 1H), 7.08 (d, *J* = 8.0 Hz, 1H), 3.79 (s, 2H), 3.47–3.43 (m, 6H), 2.75 (s, 3H), 2.64 (br s, 2H), 2.44 (s, 3H), 1.47 (s, 9H). IR (KBr): 3190, 2964, 1698, 1413, 1417, 1364, 1126, 806 *ν*_max_/cm^−1^. MS (ESI) *m/z*: 372 [M + H]^+^.

#### 2.1.3. Preparation of 1-(2,6-dimethyl-1H-indol-3-yl)-2-(piperazin-1-yl)ethan-1-one (7)

Two hundred and fifteen milligrams of **6** (0.58 mmol, 1 eq) were dissolved in dichloromethane dry. The resulting solution was cooled at 0 °C and 0.69 mL of trifluoroacetic acid (9.28 mmol, 16 eq) was added. After 3 h, the reaction was worked up adding NaOH 2 M solution until pH = 12. Then, NaCl solid was added and the solution was extracted with THF (x2). The combined organic phases were dried on sodium sulphate. After evaporation of the solvent, the crude was purified by column chromatography using EtOAc/MeOH 9:1 and MeCN/NH_3_ 9:1 as eluants to give 103 mg of the product as yellowish solid: yield: 65%; m.p.: 232.9–233.6 °C; ^1^H NMR (300 MHz, DMSO-d_6_) *δ* 11.79 (br s, NH), 7.85 (d, *J* = 7.9 Hz, 1H), 7.15 (s, 1H), 6.95 (d, *J* = 8.2 Hz, 1H), 3.83(br s, 4H), 3.62 (s, 2H), 2.86 (br s, 4H), 2.67 (s, 3H), 2.38 (s, 3H); IR (KBr): 3446, 3181, 2813, 1634, 1455, 1330, 821 *ν*_max_/cm^−1^; MS (ESI) *m/z*: 272 [M + H]^+^.

#### 2.1.4. General procedure for the synthesis of final compounds 8, 11–22

1-(2,6-Dimethyl-1*H*-indol-3-yl)-2-(piperazin-1-yl)ethan-1-one (**7**) (1 eq) was dissolved in dichloromethane dry. To the resulting solution EDCI (1 eq), TEA (2 eq), DMAP (0.1 eq) and the appropriate carboxylic acid (1 eq) were sequentially added. The reaction was stirred under nitrogen at room temperature overnight. Evaporation of the solvent gave a crude which was directly purified by column chromatography.

#### 2.1.5. 2-(4-Benzoylpiperazin-1-yl)-1-(2,6-dimethyl-1H-indol-3-yl)ethan-1-one (8)

Yellow solid; yield 29%; column eluants: EtOAc, EtOAc/MeOH 9:1; m.p.: 213.8–214.3 °C. ^1^H NMR (300 MHz, DMSO-d_6_) *δ* 11.72 (br s, NH), 7.85 (d, *J* = 8.2 Hz, 1H), 7.46–7.40 (m, 5H), 7.15 (s, 1H), 6.96 (d, *J* = 7.9 Hz, 1H), 3.67 (br s, 4H), 3.18 (br s, 2H) 2.67 (s, 3H), 2.61–2.56 (m, 4H), 2.38 (s, 3H); ^13^C NMR (75 MHz, DMSO-d_6_) *δ* 192.8, 169.5, 144.7, 136.5, 135.7, 131.5, 130.0, 129.0, 127.5, 125.1, 123.4, 121.2, 112.9, 111.6, 66.9, 53.2, 53.1, 21.7, 15.7; IR (KBr) 3189, 2990, 2828, 1609, 1446, 1282, 807 *ν*_max_/cm^−1^. MS (ESI) *m/z*: 374 [M–H]^+^. Anal. Calcd. for C_23_H_25_N_3_O_2_: C, 73.57; H, 6.71; N, 11.19; found C, 73.76; H, 6.94; N, 10.85.

#### 2.1.6. 2-(4-(4-Chlorobenzoyl)piperazin-1-yl)-1-(2,6-dimethyl-1H-indol-3-yl)ethan-1-one (11)

Yellow solid; yield 33%; column eluants: EtOAc, EtOAc/MeOH 9:1; m.p.: 240.7–241.3 °C; ^1^H NMR (300 MHz, DMSO-d_6_) *δ* 11.70 (br s, NH), 7.85 (d, *J* = 7.9 Hz, 1H), 7.50 (br d, AA’XX’, 2H), 7.42 (br d, AA’XX’, 2H), 7.14 (s, 1H), 6.95 (d, *J* = 8.2 Hz, 1H), 3.67 (br s, 4H), 3.33 (br s), 2.67 (s, 3H), 2.61–2.56 (m, 4H), 2.38 (s, 3H); ^13^C NMR (75 MHz, DMSO-d_6_) *δ* 193.17, 168.4, 144.8, 135.7, 135.2, 134.8, 131.5, 129.5, 129.1, 125.1, 123.5, 121.2, 112.7, 111.6, 53.14, 53.14, 52.9, 21.7, 15.7. IR (KBr): 3225, 2793, 2358, 1609, 1442, 1261, 864 *ν*_max_/cm^−1^. MS (ESI) *m/z*: 410 [M + H]^+^. Anal. Calcd. for C_23_H_24_ ClN_3_O_2_: C, 67.39; H, 5.90; N, 10.25; found C, 67.11; H, 6.12; N, 10.54.

#### 2.1.7. 1-(2,6-Dimethyl-1H-indol-3-yl)-2-(4-(4-methoxybenzoyl)piperazin-1-yl)ethan-1-one (12)

Yellow solid; yield 53%; column eluants: EtOAc, EtOAc/MeOH 9:1; m.p.: 219.9–220.8 °C.; ^1^H NMR (300 MHz, DMSO-d_6_) *δ* 11.70 (br s, NH), 7.85 (d, *J* = 8.2 Hz, 1H), 7.38 (br d, AA’XX’, 2H), 7.14 (s, 1H), 6.99–6.94 (m, 3H), 3.79 (br d, 3H), 3.67 (br s, 2H), 3.51 (br s, 4H) 2.67 (s, 3H), 2.58 (br s, 4H), 2.38 (s, 3H); ^13^C NMR (75 MHz, DMSO-d_6_) *δ* 192.8, 169.5, 144.6, 136.5, 135.7, 131.5, 130.0, 129.0, 127.5, 125.1, 123.4, 121.2, 112.8, 111.6, 66.9, 55.8, 53.2, 53.1, 21.7, 15.7; IR (KBr): 3235, 3003, 2807, 1613, 1463, 1253, 977 *ν*_max_/cm^−1^; MS (ESI) *m/z*: 406 [M + H]^+^. Anal. Calcd. for C_24_H_27_N_3_O_3_: C, 71.09; H, 6.71; N, 10.36; found C, 71.10; H, 6.75; N, 10.32.

#### 2.1.8. 4-(4-(2-(2,6-Dimethyl-1H-indol-3-yl)-2-oxoethyl)piperazine-1-carbonyl)benzonitrile (13)

Yellow solid; yield 23%; column eluants: EtOAc, EtOAc/MeOH 9:1; m.p.: 243.9–244.8 °C. ^1^H NMR (300 MHz, DMSO-d_6_) *δ* 11.70 (br s, NH), 7.92–7.84 (m, 3H), 7.58 (br d, AA’XX’, 2H), 7.15 (s, 1H), 6.96 (br d, 1H), 3.68 (br s, 4H), 3.29 (br s, 2H), 2.67 (s, 3H), 2.51 (br s, 4H), 2.38 (s, 3H); ^13^C NMR (75 MHz, DMSO-d_6_) *δ* 192.5, 166.3, 143.2, 139.4, 134.1, 131.6, 129.9, 126.8, 123.5, 121.9, 119.6, 117.4, 111.2, 111.1, 110.1, 65.1, 51.7, 51.2, 20.1, 14.1; IR (KBr): 3410, 3254, 2816, 2790, 2233, 1609, 1454, 1291, 979 *ν*_max_/cm^−1^; MS (ESI) *m/z*: 401[M + H]^+^. Anal. Calcd. for C_24_H_24_N_4_O_2_: C, 71.98; H, 6.04; N, 13.99; found C, 72.13; H, 6.23; N, 14.08.

#### 2.1.9. 1-(2,6-Dimethyl-1H-indol-3-yl)-2-(4-(thiophene-2-carbonyl)piperazin-1-yl)ethan-1-one (14)

Yellow solid; yield 29%; column eluants: EtOAc, EtOAc/MeOH 9:1; m.p.: 200.3–201.2 °C; ^1^H NMR (300 MHz, DMSO-d_6_) *δ* 11.74 (br s, NH), 7.86 (d, *J* = 8.2 Hz, 1H), 7.76 (br d, 1H), 7.41–7.40 (m, 1H), 7.15–7.10 (m, 2H), 6.96 (d, *J* = 8.2 Hz, 1H), 3.75–3.67 (m, 4H), 3.37 (br s, 2H) 2.68 (s, 3H), 2.62 (s, 4H), 2.38 (s, 3H); ^13^C NMR (75 MHz, DMSO-d_6_) *δ* 192.8, 167.8, 144.7, 137.8, 135.7, 131.5, 130.0, 129.6, 127.7, 125.1, 124.1, 123.4, 121.2, 112.8, 111.6, 66.7, 53.3, 21.7, 15.7; IR (KBr): 3270, 2927, 2793, 1642, 1454, 1261, 809 *ν*_max_/cm^−1^; MS (ESI) *m/z*: 382[M + H]^+^; Anal. Calcd. for C_21_H_23_N_3_O_2_S: C, 66.12; H, 6.08; N, 11.01; found C, 66.23; H, 6.26; N, 10.93.

#### 2.1.10. 1-(2,6-Dimethyl-1H-indol-3-yl)-2-(4-nicotinoylpiperazin-1-yl)ethan-1-one (15)

Yellow solid; yield 39%; column eluants: EtOAc, EtOAc/MeOH 9:1; m.p.: 216.2–216.8 °C; ^1^H NMR (300 MHz, DMSO-d_6_) *δ* 11.70 (br s, NH), 8.65–8.61 (m, 2H), 7.87–7.82 (m, 2H), 7.50–7.45 (m, 1H), 7.15 (s, 1H), 6.95 (br d, 1H), 3.68 (br s, 4H), 3.37 (br s, 2H), 2.67 (s, 3H), 2.56 (br s, 4H), 2.37 (s, 3H); ^13^C NMR (75 MHz, DMSO-d_6_) *δ* 192.6, 167.2, 151.0, 148.1, 144.7, 135.7, 135.4, 132.3, 131.5, 125.1, 124.1, 123.5, 121.2, 116.2, 111.6, 66.7, 53.4, 52.9, 21.7, 15.7; IR (KBr): 3414, 3213, 2828, 1621, 1454, 1267, 1301, 817 *ν*_max_/cm^−1^; MS (ESI) *m/z*: 377 [M + H]^+^; Anal. Calcd. for C_22_H_24_N_4_O_2_: C, 70.19; H, 6.43; N, 14.88; found C, 70.21; H, 6.44; N, 14.73.

#### 2.1.11. 1-(2,6-Dimethyl-1H-indol-3-yl)-2-(4-(2-methylbenzoyl)piperazin-1-yl)ethan-1-one (16)

Yellow solid; yield 41%; column eluants: EtOAc, EtOAc/MeOH 9:1; m.p.: 200.5–201.6 °C; ^1^H NMR (300 MHz, DMSO-d_6_) *δ* 11.70 (br s, NH), 7.85 (d, *J* = 8.2 Hz, 1H), 7.31–7.27 (m, 3H), 7.26–7.24 (m, 2H), 7.15 (d, 1H), 3.69 (br s, 2H), 3.34 (br s, 2H), 3.15 (br s, 2H), 2.67 (s, 3H), 2.51 (br s, 4H), 2.44 (s, 3H), 2.38 (s, 3H); ^13^C NMR (75 MHz, DMSO-d_6_) *δ* 192.6, 169.0, 144.7, 135.7, 134.2, 131.8, 131.5, 130.7, 129.2, 126.4, 126.2, 123.4, 121.2, 112.8, 66.8, 53.5, 53.0, 46.8, 41.4, 21.7, 19.2, 15.7; IR (KBr): 3431, 3221, 2919, 2797, 1615, 1454, 1257, 748 *ν*_max_/cm^−1^; MS (ESI) *m/z*: 390 [M + H]^+^. Anal. Calcd. for C_24_H_27_N_3_O_2_: C, 74.01; H, 6.99; N, 10.79; found C, 74.01; H, 7.01; N, 10.63.

#### 2.1.12. 1-(2,6-Dimethyl-1H-indol-3-yl)-2-(4-(3-methoxybenzoyl)piperazin-1-yl)ethan-1-one (17)

Yellow solid; yield 32%; column eluants: EtOAc, EtOAc/MeOH 9:1; m.p.: 209.8–210.4 °C; ^1^H NMR (300 MHz, DMSO-d_6_) *δ* 11.71 (br s, NH), 7.85 (d, *J* = 7.9 Hz, 1H), 7.34 (t, 1H), 7.15 (br s, 1H), 7.02–6.92 (m, 4H), 3.78 (s, 3H), 3.68 (br s, 3H), 3.35 (br s, 3H), 2.67 (br s, 7H), 2.38 (s, 3H); ^13^C NMR (75 MHz, DMSO-d_6_) *δ* 192.7, 169.1, 159.7, 144.7, 137.9, 135.7, 131.5, 130.2, 125.1, 123.4, 121.2, 119.4, 115.7, 112.7, 111.6, 66.8, 55.8, 53.3, 53.11, 47.4, 21.7, 15.7; IR (KBr): 3131, 3049, 2944, 1651, 1455, 1292, 1130, 968 *ν*_max_/cm^−1^; MS (ESI) *m/z*: 406 [M + H]^+^. Anal. Calcd. for C_24_H_27_N_3_O_3_: C, 71.09; H, 6.71; N, 10.36; found C, 70.85; H, 6.45; N, 10.76.

#### 2.1.13. 2-(4-(3,4-Difluorobenzoyl)piperazin-1-yl)-1-(2,6-dimethyl-1H-indol-3-yl)ethan-1-one (18)

Yellow solid; yield 27%; column eluants: EtOAc, EtOAc/MeOH 9:1; m.p.: 233.8–235.0 °C; ^1^H NMR (300 MHz, DMSO-d_6_) *δ* 11.71 (br s, NH), 7.85 (d, *J* = 7.9 Hz, 1H), 7.56–7.47 (m, 2H), 7.28 (br s, 1H), 7.15 (s, 1H), 6.96 (d, *J* = 7.9 Hz, 1H), 3.68 (br s, 2H), 3.33 (br s, 2H), 2.67 (s, 3H), 2.51 (br s, 6H), 2.38 (s, 3H); MS (ESI) *m/z*: 412 [M + H]^+^; IR (KBr): 3252, 2919, 2795, 1618, 1469, 1286, 1046, 980 *ν*_max_/cm^−1^; MS (ESI) 412 [M + H]^+^. Anal. Calcd. for C_23_H_23_F_2_N_3_O_2_: C, 67.14; H, 5.63; N, 10.21; found C, 67.43; H, 5.79; N, 10.59.

#### 2.1.14. 2-(4-(3-Chlorobenzoyl)piperazin-1-yl)-1-(2,6-dimethyl-1H-indol-3-yl)ethan-1-one (19)

Yellow solid; yield 32%; column eluants: EtOAc, EtOAc/MeOH 9:1; m.p.: 222.3–223.5 °C; ^1^H NMR (300 MHz, CDCl_3_) *δ* 9.42 (br s, NH), 7.71 (d, *J* = 7.9 Hz, 1H), 7.40–7.25 (m, 4H), 7.04 (d, *J* = 8.2 Hz, 2H), 3.85 (br s, 4H), 3.47 (br s, 2H), 2.80 (br s, 1H), 2.68 (br s, 6H), 2.40 (s, 3H); ^13^C NMR (75 MHz CDCl_3_,) *δ* 192.5, 169.0, 144.7, 137.5, 135.3, 134.7, 132.3, 130.0, 127.3, 125.2, 124.0, 123.7, 120.6, 112.8, 111.4, 67.0, 53.7, 53.7, 29.6, 21.5, 15.7; IR (KBr): 3264, 2916, 2795, 1646, 1454, 1256, 978, 809 *ν*_max_/cm^–1^; MS (ESI) *m/z*: 410 [M + H]^+^. Anal. Calcd. for C_23_H_24_ClN_3_O_2_: C, 67.39; H, 5.90; N, 10.25; found C, 67.38; H, 5.90; N, 10.24.

#### 2.1.15. 1-(2,6-Dimethyl-1H-indol-3-yl)-2-(4-(4-methylbenzoyl)piperazin-1-yl)ethan-1-one (20)

Yellow solid; yield 48%; column eluants: EtOAc, EtOAc/MeOH 9:1; m.p.: 230.9–231.2 °C; ^1^H NMR (300 MHz, DMSO-d_6_) *δ* 11.71 (br s, NH), 7.86–7.82 (m, 1H), 7.31–7.23 (m, 4H), 7.15 (s, 1H), 6.96 (d, *J* = 7.9 Hz, 1H), 3.69 (br s, 2H), 3.35 (br s, 4H), 2.67 (s, 3H), 2.51. IR (KBr): 3228, 2915, 2792, 1607, 1454, 1260, 979 *ν*_max_/cm^−1^; MS (ESI) *m/z*: 390 [M + H]^+^; Anal. Calcd. for C_24_H_27_N_3_O_2_: C, 74.01; H, 6.99; N, 10.79; found C, 74.12; H, 7.02; N, 10.79.

#### 2.1.16. 2-(4-(Cyclopentanecarbonyl)piperazin-1-yl)-1-(2,6-dimethyl-1H-indol-3-yl)ethan-1-one (21)

Brown oil; yield 61%; column eluants: EtOAc, EtOAc/MeOH 9:1; ^1^H NMR (300 MHz, CDCl_3_) *δ* 9.00 (br s, NH), 7.73 (d, *J* = 8.2 Hz, 1H), 7.26 (br d, 1H), 7.12 (s, 1H), 7.05 (d, *J* = 8.2 Hz, 1H), 3.83 (s, 2H), 3.75 (br s, 2H), 3.63 (br s, 2H), 2.80–2.71 (m, 7H), 2.45 (s, 3H), 1.92–1.46 (m, 7H); IR (KBr): 3253, 2944, 2862, 1650, 1620, 1455, 1234, 957 *ν*_max_/cm^−1^; MS (ESI) *m/z*: 368 [M + H]^+^; Anal. Calcd. for C_22_H_29_N_3_O_2_: C, 71.90; H, 7.95; N, 11.43; found C, 72.23; H, 8.31; N, 11.32.

#### 2.1.17. 1-(4-(2-(2,6-Dimethyl-1H-indol-3-yl)-2-oxoethyl)piperazin-1-yl)heptan-1-one (22)

Brown oil; yield 76%; column eluants: EtOAc, EtOAc/MeOH 9:1; ^1^H NMR (300 MHz, CDCl_3_) *δ* 10.25 (br s, NH), 7.68 (d, *J* = 8.2 Hz, 1H), 7.08 (s, 1H), 7.00 (d, *J* = 7.9 Hz, 1H), 3.83 (s, 2H), 3.73 (br s, 2H), 3.56 (br s, 2H), 2.77–2.71 (m, 3H), 2.62 (s, 3H), 2.38 (s, 3H), 2.32–2.27 (m, 5H), 1.59–1.54 (m, 2H), 1.26 (br s, 4H), 0.85 (br t, 3H); ^13^C NMR (75 MHz, CDCl_3_) *δ* 191.7, 172.4, 145.4, 135.5, 132.1, 124.0, 123.6, 120.4, 112.4, 111.6, 66.2, 53.3, 53.1, 45.3, 41.2, 34.5, 31.6, 28.9, 25.0, 22.5, 21.5, 15.6, 14.1; IR (KBr): 2927, 2857, 1731, 1645, 1455, 1434, 1234, 668 *ν*_max_/cm^−1^; MS (ESI) *m/z*: 384 [M + H]^+^; Anal. Calcd. for C_23_H_33_N_3_O_2_: C, 72.03; H, 8.67; N, 10.96; found C, 72.03; H, 8.73; N, 11.21.

### 2.2. Cell lines

Madin-Darby canine kidney (MDCK) cells stably expressing One Strep Tag DGKα (OST-DGKα) were prepared by infecting MDCK cells with a vector expressing an inducible OST tagged DGKα constructs^17^. MDCK cells infected with empty vector were used as controls. MDCK cells were cultured in MEM (minimal essential medium) with 5% FBS (foetal bovine serum) and 1% antibiotic–antimycotic solution. Routinely, cells were splitted every 3–4 days with trypsin–EDTA 0.25% in standard 100 mm dishes.

Human embryonic kidney 293T cells (10 cm^2^ plates) were cultured in RPMI with 10% FBS and 1% penicillin/streptomycin and cultures were maintained by splitting them for every 2–3 days using trypsin–EDTA 0.25%.

Michigan Cancer Foundation 7 (MCF7) cells were cultured in DMEM with 10% FBS + 1% penicillin/streptomycin and cultures were maintained by splitting them for every 2–3 days using trypsin–EDTA 0.25%.

### 2.3. Primary cells

PBL were isolated from healthy anonymous human donors by Ficoll-Paque PLUS (GE Healthcare, Chicago, IL) density gradient centrifugation, washed, and resuspended at 2 × 10^6^ cell/mL in RPMI-GlutaMAX containing 10% heat inactivated FCS, 2 mM glutamine, and 100 U/mL of penicillin and streptomycin. T cells were activated with 1 µg/mL anti-CD3 (UCHT1) and anti-CD28 (clone CD28.2) antibodies. After three days, activated T cells were washed and cultured in medium additionated of 100 IU/mL rhIL-2 (Peprotech, Rocky Hill, NJ) at 1.2 × 10^6^ cells/mL for ≥7 days by changing media for every 2–3 days.

Human monocytes were isolated from healthy anonymous human buffy coats (provided by the Transfusion Service of Ospedale Maggiore della Carità, Novara, Italy) by the standard technique of dextran sedimentation and Histopaque (density = 1.077 g cm^3^, Sigma-Aldrich, Milano, Italy) gradient centrifugation (400×*g*, 30 min, room temperature) and recovered by fine suction at the interface, as described previously^32^. Purified monocytes populations were obtained by adhesion (90 min, 37 °C, 5% CO_2_) in serum-free RPMI 1640 medium (Sigma-Aldrich, Milano, Italy) supplemented with 2 mM glutamine and antibiotics. Cell viability (trypan blue dye exclusion) was usually >98%.

### 2.4. Preparation of DGKα enriched homogenates

Large cultures of MDCK cells for enzyme preparation were done by plating 5 × 10^6^ cells in 245 mm^2^ dishes. Once they reached nearly 70% confluence, cells were treated with doxycycline (1 µg/mL, two days). After two days of treatment, each plate was washed in cold PBS and cells homogenised in 5 mL of homogenate buffer (25 mM Hepes (pH 8), 20% glycerol, 135 mM NaCl, 5 mM ethylenediaminetetraacetic acid (EDTA), 1 mM ethylene glycol-bis(beta-aminoethyl ether)-N,N,N′,N′-tetraacetic acid (EGTA), 1 mM sodium orthovanadate, and protease inhibitor cocktail) for each dish. Cells were collected with a rubber scraper, homogenised by passing them through a 29 G-needle syringe 20 times and stored in aliquots at –80 °C. Presence of OST-DGKα was confirmed by western blotting and enzyme assay, transduced DGKα has an activity >100 folds the endogenous DGK.

### 2.5. Preparation of DGKζ and DGKθ enriched homogenates

293T cells were transiently transfected with indicated DGK isoform plasmid DNA using Lipofectamine 3000, Invitrogen (Carlsbad, CA). Forty eight hours after transfection, cells were harvested and homogenised with a 29 G-needle using 500 µL of homogenate buffer for each dish and immediately stored in aliquots at –80 °C. Cells transfected with empty vector were used as controls, overexpressed DGK has an activity >50 folds the endogenous.

### 2.6. DGK assay

Essentionally, the same procedure was followed as reported previously in Velnati et al.^9^ In brief, DGK activity was assayed by measuring initial velocities (5 min at 27 °C) in presence of saturating substrate concentrations. Reaction conditions: 0.9 µg/µL 1,2-dioleoyl-sn-glycerol, 5 mM ATP, 0.01 µCi/µL [γ^32^P]-ATP, 1 mM sodium orthovanadate, 10 mM MgCl_2_, 1.2 mM EGTA in 7.5 mM Hepes pH 8^12^. Reaction mixture is assembled mixing enzyme (24.5 µL of homogenate), 100× inhibitor or DMSO (0.5 µL), 5× ATP solution (10 µL of 25 mM ATP, 0.05 µCi/µL [γ^32^P]-ATP (Perkin-Elmer, Milan, Italy), 5 mM sodium orthovanadate, 50 mM MgCl_2_), and 3.3× DAG solution (15 µL of 3 µg/µL 1,2-dioleoyl-sn-glycerol resuspended by sonication in 4 mM EGTA in 25 mM Hepes pH 8). The reaction was stopped after 5 min by adding 200 µL of freshly prepared 1 M HCl and lipid was extracted by adding 200 µL of CH_3_OH:CHCl_3_ 1:1 solution and vortexing for 1 min. The two phases were separated by centrifugation (12,000 RCF for 2 min). Twenty-five microlitres of the lower organic phase was spotted in small drops on silica TLC plates. TLC was run 10 cm and dried before radioactive signals were detected by GS-250 molecular imager and was quantified by quantity one (Bio-Rad, Hercules, CA) software assuring the absence of saturated spots.

Percentage residual activity was calculated as follows: (OST-DGKα homogenate with inhibitor – vector homogenate)/(OST-DGKα homogenate with DMSO – vector homogenate)×100.

### 2.7. Superoxide anion (O^2−^) production

All the experiments were performed in triplicate using cells isolated from each single donor.

Monocytes (250,000 cells/well) were treated for 1 h with the indicated drugs (10 µM) with or without serotonin (1 µM). Then, cells were stimulated with phorbol 12-myristate 13-acetate (PMA; Sigma-Aldrich, Milano, Italy) 1 µM for 30 min. PMA is a well-known stimulus that induces a strong and significant respiratory burst via PKC activation^33^. Superoxide anion production was then evaluated by the superoxide dismutase (SOD)-sensitive cytochrome C (CytC) reduction assay and expressed as nmoles CytC reduced/10^6^ cells/30 min, using an extinction coefficient of 21.1 mM. To avoid interference with spectrophotometrical recordings, cells were incubated with RPMI 1640 without phenol red, antibiotics, and FBS.

### 2.8. RICD assay in SAP silenced T cells

Activated human PBLs were transfected with 200 pmol of siRNA oligonucleotides specific for the target protein (Stealth Select siRNA; Life Technologies, Carlsbad, CA) or a non-specific control oligo (Life Technologies, Carlsbad, CA). Transient transfections were performed using Amaxa nucleofector kits for human T cells (Lonza, Basel, Switzerland) and the Amaxa Nucleofector II or 4D systems (programmes T-20 or EI-115). Cells were cultured in IL-2 (100 IU/mL) for four days to allow target gene knockdown. Knockdown efficiency was periodically evaluated by Western blotting.

Non-specific Stealth RNAi Negative Control Duplexes (12935-300, Life Technologies, Carlsbad, CA) were used as a negative control.

siRNA SAP: sense strand UGUACUGCCUAUGUGUGCUGUAUCA, antisense strand UGAUACAGCAGACAUAGGCAGUACA.

To test restimulation induced cell death, activated T cells (10^5^ cells/well) were plated in triplicate in 96-well round-bottom plate and treated with anti-CD3 (clone OKT3) (10 ng/mL) in RPMI-GlutaMAX supplemented with 100 IU/mL rhIL-2 for 24 h. In these assays, inhibitors (10 µM) were added 30 min before the restimulation with OKT3. 24 h after treatment, cells were stained with 20 ng/mL propidium iodide and collected for a constant time of 30 s per sample on Attune Nxt Flow Cytometer (Thermo Fisher Scientific, Waltham, MA). Cell death is expressed as % cell loss and calculated as:

$$\%\;\text{cell}\;\text{loss}=\left(1-\left(\frac{\text{number}\;\text{of}\;\text{viable}\;\text{cells}\;\text{in}\;\text{sample}}{\text{number}\;\text{of}\;\text{viable}\;\text{cells}\;\text{in}\;\text{control}}\right)\times 100\right)$$

Results were expressed as mean ± standard error of the mean (SEM). We always compared controls and SAP silenced lymphocytes from the same donors as there is a large individual variability in RICD sensitivity.

### 2.9. Migration assays

Cell migration assays were performed using the Culture-Insert 2 well in µ-Dish (ibidi GmbH, Martinsried, Germany).

Briefly, 25,000 MCF7 cells were plated in each well and cultured for 24 h. The day after, the culture insert was removed and the cells were washed with PBS before treating them with respective DGKα inhibitors (10 µM) or DMSO for 15 h in complete medium (DMEM 10% FBS + 1% penicillin/streptomycin), while medium without FBS was used as a negative control for migration.

Phase-contrast pictures were taken immediately after treatment (0 h) and after 15 h under 5× magnification.

Finally, wound areas were determined using ImageJ software (NIH, , Bethesda, MD). Wound reduction was calculated by using the following formula: (wound area at 15 h/wound at 0 h)×100, the values obtained were expressed as the percentage of wound area compared to the initial area.

### 2.10. Quantification and statistical analysis

Data for the screen on OST-DGKα homogenates are the mean of duplicates. The compounds showing inhibitory activity in this assay were tested >4 times and the mean ± SEM is reported.

To calculate IC_50_ values of active inhibitors, the inhibitor activity was measured at least three times at 0.1, 1.0, 10.0, and 100.0 µM concentration. Data were analysed using [inhibitor] vs. normalised response parameters with least square [ordinary] curve fitting method in GraphPad PRISM 8.0 software (GraphPad Software, La Jolla, CA) mentioning 95% confidence interval and IC_50_ values always greater than 0.0. Graph shows the mean ± SEM of inhibitor activity at the indicated concentration. In all the experiments, the data were normalised with the controls.

Evaluation of *in vitro* assays across multiple treatments (RICD), SOD-sensitive CytC reduction assay, migration assays were analysed by using one-way ANOVA with multiple comparisons correction using GraphPad PRISM 8.0 software (GraphPad Software, La Jolla, CA). Error bars are described in figure legends as ± SEM or ± SD where appropriate. A single, double, triple and four asterisk denotes significance of a *p* value ≤0.05, ≤0.01, ≤0.001, and ≤0.0001 respectively in all experiments.

### 2.11. Pharmacophoric model

A representative 3D structure of each compound was generated using OMEGA2 software^34–36^. The generated file was used to generate a pharmacophore model with the Pharmagist web server (bioinfo3d.cs.tau.ac.il/PharmaGist/)^37^.

## 3. Results

### 3.1. Chemistry

**Figure 3.**
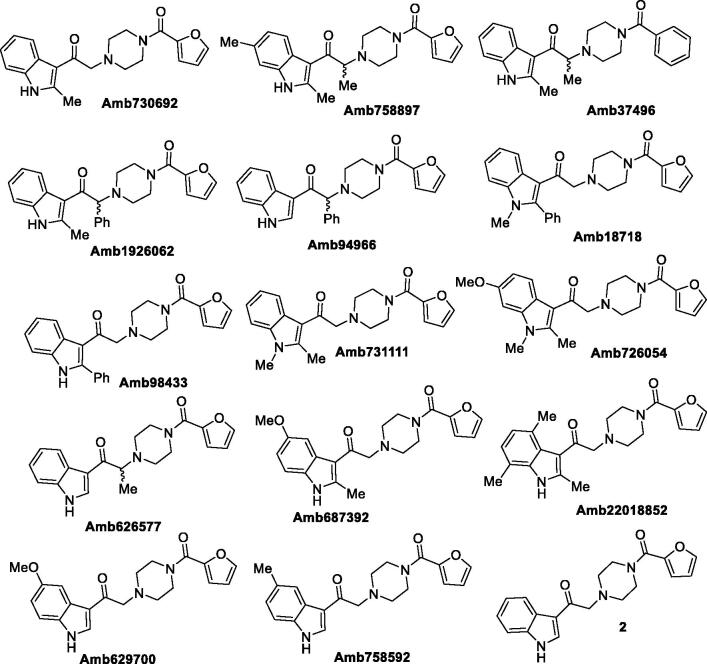
First set of compounds tested for their inhibitory activity on DGKα.

At the beginning, we purchased 14 analogues of Amb639752 by vendors (Figure 3), while one analogue (**2**), being not commercially available, was synthesised (see Supplementary material). All the compounds were evaluated for their inhibitory activity on DGKα at a concentration of 100 µM (Table 1).

**Table 1.** Inhibitory activity on DGKα (I).

Compound	Residue activity at 100 µM
R59022	27
R59949	28
Amb639752	4
Amb758897	67
Amb37496	114
Amb626577	127
Amb1926062	88
Amb94966	196
Amb730692	45
Amb98433	81
Amb18718	135
Amb726054	126
Amb731111	130
Amb22018852	160
Amb687392	115
Amb758592	150
Amb629700	101
**2**	135

Each inhibitor was tested in duplicate at least once, and DGKα activity was expressed as percentage of residual DGKα activity compared to DMSO control in the same assay. Assay uses OST-DGKα overexpressing cell lysates in presence of saturating exogenous DAG and ATP. We considered R59022 (commercially available) and the lead compound Amb63975230 as our reference molecules. As expected, our reference inhibitors R59022 and Amb639752 featured 73% and 96% inhibition respectively, confirming the quality of data obtained.

This first screening showed us how Amb639752 exhibits a rigid structure activity relationship. Indeed, both the methyl groups on the 2,6 position of indole are mandatory, the NH indole cannot be alkylated as well as ramifications on the alkyl chain are detrimental. We then focussed our attention on furan ring knowing its intrinsic toxicity via metabolic activation^38^. Unfortunately, there were no analogues available by vendors. Our first goal was to replace the furan moiety with the phenyl ring, investigating two different synthetic pathways.

**Scheme 1.**
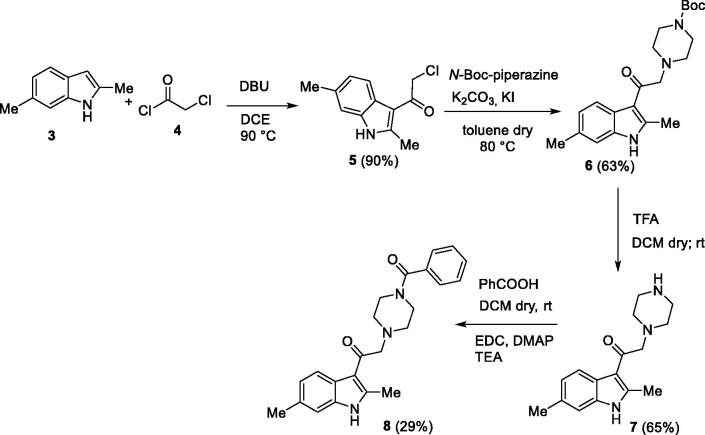
The first synthetic route for the compound **8**.

In the first one, the commercially available 2,6-dimethyl-1*H*-indole **3** was acylated with 2-chloroacetyl chloride **4**, in the presence of DBU in dichloroethane^39^ to give the derivative **5** 90% yield. Then, the acylated compound **5** was reacted with *N*-Boc piperazine in the presence of potassium carbonate and potassium iodide to afford the piperazinic derivative **6** in 63% yield. Boc deprotection with trifluoroacetic acid, followed by coupling with benzoic acid using the condensing agent EDCI afforded the final compound **8** (Scheme 1).

**Scheme 2.**
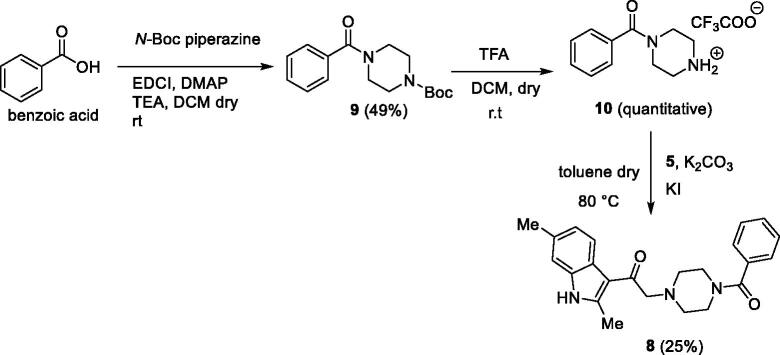
The second synthetic route for the compound **8**.

In the second synthetic strategy, we initially coupled the benzoic acid with *N*-Boc piperazine in the presence of EDCI to give piperazinic derivative **9** in 49% yield. Boc deprotection gave in quantitative yield the compound **10**. Due to its high aqueous solubility, solvent was evaporated and the crude as trifluoroacetate salt was directly used for the next step, where it was reacted with the acylated indole **5** to give the final compound **8** in 25% yield (Scheme 2) (see Supplementary material for full synthetic details).

**Figure 4.**
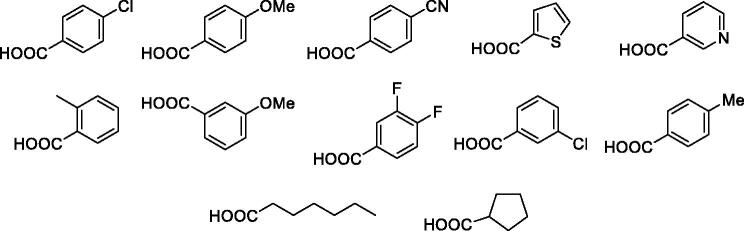
Carboxylic acids used.

**Figure 5.**
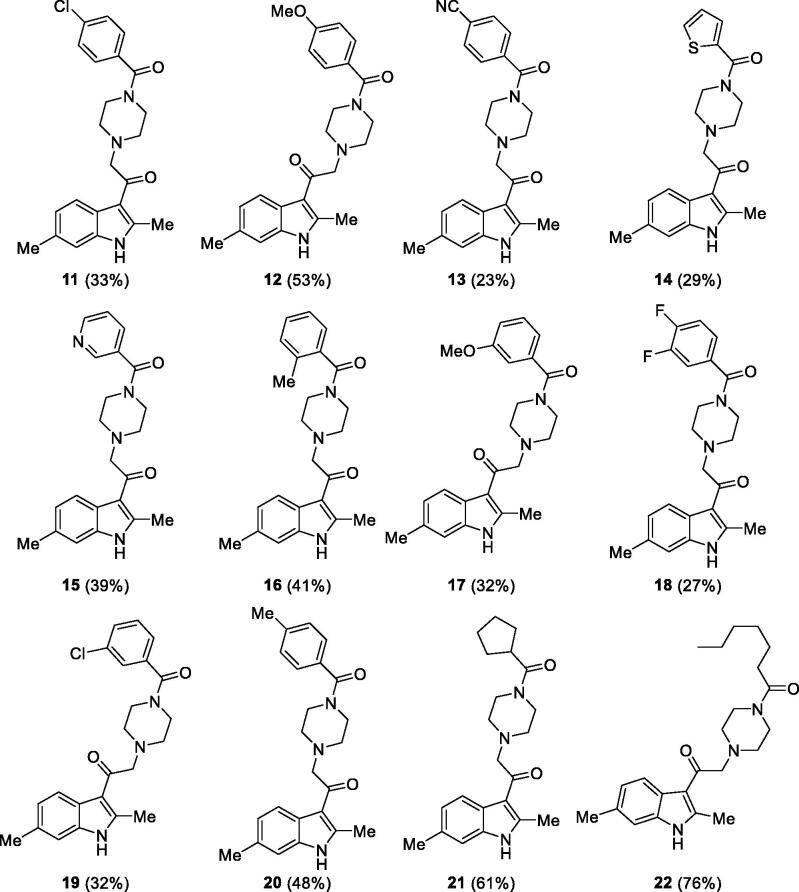
Putative DGKα inhibitors synthesised. In brackets the yield of the coupling reaction with the common intermediate **7**.

Overall yield calculation was 11% for both strategies, but with the first route it was possible to use a common synthetic intermediate **7** which can be coupled with different carboxylic acids. Furthermore, the second route requires more purification steps. For this reason, we applied the first route and coupled the advanced intermediate **7** with 12 different carboxylic acids (Figure 4) to afford compounds **11**–**22** (Figure 5).

All those molecules were dissolved in DMSO and tested at a concentration of 100 µM for the ability to inhibit DGKα using equal amounts of DMSO as control. We identified eight compounds capable of reducing OST-DGKα activity similar or superior to R59022 (Table 2).

**Table 2.** Inhibitory activity on DGKα (II).

Compound	Residue activity at 100 µM	IC_50_ (µM)
**8**	5	3.2
**11**	6	1.6
**12**	6	3.6
**13**	14	6.9
**14**	6	3.0
**15**	89	–
**16**	27	32.8
**17**	41	–
**18**	47	–
**19**	26	49.7
**20**	3	1.8
**21**	39	–
**22**	48	–

### 3.2. Potency and isoform specificity of active molecules

To measure the inhibitor potency, we determined the IC_50_ values for the compounds that resulted active when tested at 100 µM by measuring the residual OST-DGKα activity over a dose range of inhibitor concentrations (0.1 µM, 1.0 µM, 10.0 µM, and 100.0 µM).

**Figure 6.**
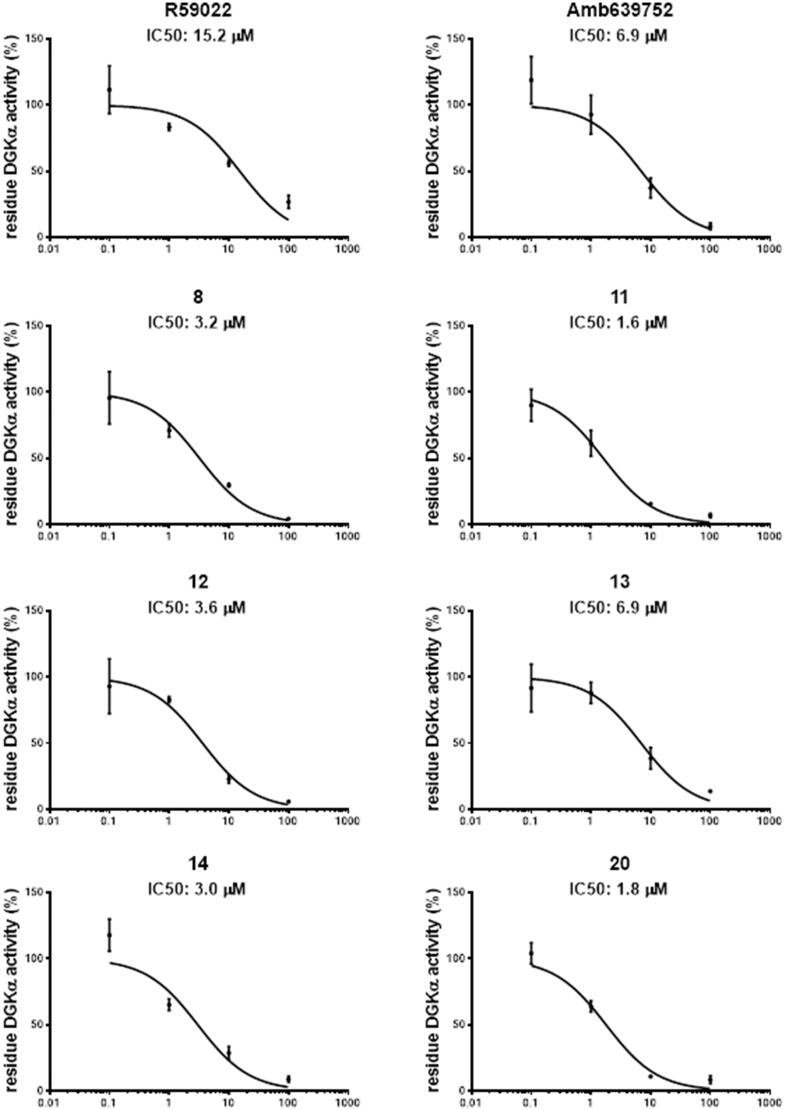
Dose–response curves for novel DGKα inhibitors. Dose–response of the most active compounds along with their IC_50_ values. Data from at least three independent experiments performed in triplicate.

For R59022 and Amb639752, we measured IC_50_ values of 15.2 ± 5.8 µM and 6.9 ± 3.0 µM respectively which were comparable to previous reports using similar assay conditions^9^. Considering those two as reference/template compounds, we measured the IC_50_ values of **8**, **11**, **12**, **13**, **14**, **16**, **19**, and **20** as 3.2 ± 1.0, 1.6 ± 0.4, 3.6 ± 1.2, 6.9 ± 2.3, 3.0 ± 1.0, 32.8 ± 11.5, 49.7 ± 31.7, and 1.8 ± 0.4 µM, respectively, signifying that their activity is equal or superior to the template compounds (Figure 6).

Due to their higher IC_50_ values, we thus decided to exclude **16** and **19** for further experiments. In summary, we recognised six compounds with equal or superior inhibitory activity compared to commercially available DGKα inhibitors.

**Figure 7.**
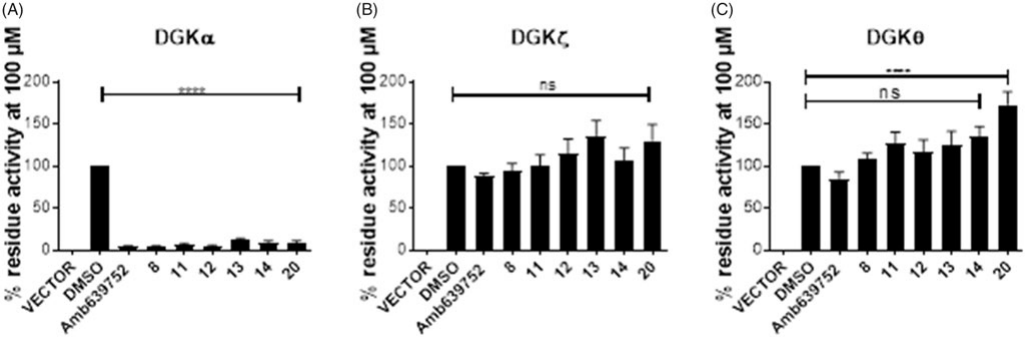
Isoform specificity of novel DGKα inhibitors. 293T cells were transfected with different DGK isoforms (A – DGKα, B – DGKζ, C – DGKθ, respectively) or empty vectors and homogenised. All the molecules were tested at 100 μM for their capacity to inhibit the DGK activity of the different isoform homogenates. Data are means ± SEM of at least three independent experiments performed in triplicate.

To check the isoform specificity of those active molecules, we tested them, along with Amb639752, for their ability to inhibit DGKα, DGKζ (the other major DGK isoform expressed in lymphocytes), and the more distantly related and widely expressed DGKθ. At the highest concentration of 100 µM, all those molecules resulted in highly specific against DGKα as like their parent molecule, Amb639752 by completely inhibit DGKα whereas they do not have significant effects on DGKζ and DGKθ apart from **20** which, at the contrary, acts as an activator of DGKθ (Figure 7).

### 3.3. Activity of compounds on serotonin receptors

R59022, R59949, and ritanserin feature a dual activity as DGKα inhibitors and serotonin receptor antagonists^25^. Conversely, Amb639752 was reported as a selective DGKα inhibitor which has no effects on serotonin activity^9^. Thus, we investigated whether the active molecules identified affect serotonin signalling.

**Figure 8.**
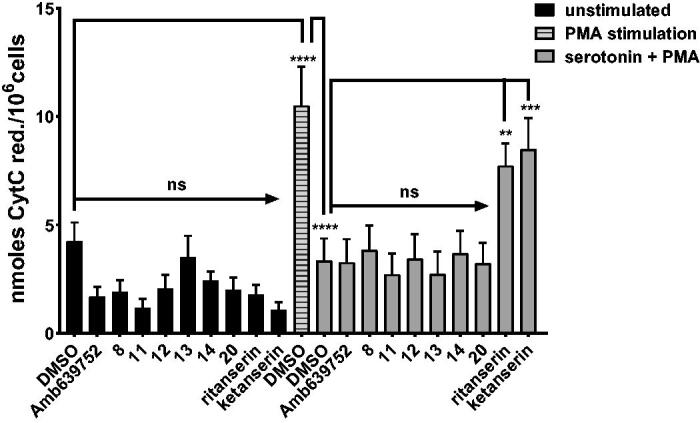
Novel DGKα inhibitors do not affect serotonin signalling. Human monocytes were pre-incubated for 1 h with the indicated drugs in absence or presence of serotonin and then stimulated with PMA 1 µM for 30 min (■ control unstimulated cells, 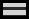 PMA stimulated cells, ■ PMA and serotonin stimulated cells). Results are expressed as n moles of reduced cytochrome C/10^6^ cells. Data are means ± SEM of 10 independent experiments performed in triplicate.

To this purpose, we measured the effect of serotonin on PMA-induced oxidative burst in human monocytes. As previously shown, 1 µM serotonin reverses the oxidative burst to control values^33^. Known serotonin receptor antagonists ritanserin and ketanserin (10 µM) impaired serotonin action, while pure DGKα inhibitors such as Amb639752 had no effect (Figure 8). These data indicate that this assay is sensitive to perturbations in serotonin signalling independently of activity against DGKα. Interestingly, as like Amb639752, all the newly synthesised active molecules did not affect serotonin action (Figure 8), indicating that all those molecules are not serotonin receptor antagonists.

### 3.4. DGKα inhibitors restore RICD in SAP deficient T cells

Ruffo et al. demonstrated that the defective RICD observed in T cells from XLP-1 patients was rescued by silencing DGKα expression or by pre-treatment with DGKα inhibitors R59949 or R59022^8^. Interestingly, R59022 also showed beneficial effects in an *in vivo* model of XLP-1, but due to its poor pharmacological proprieties, its use in human patients results unlikely. We therefore tested the effect all those active molecules along with Amb639752 on RICD sensitivity of SAP-deficient T cells. As additional controls, we also included ritanserin and ketanserin to evaluate the contribution of serotonin antagonism to the effects observed.

**Figure 9.**
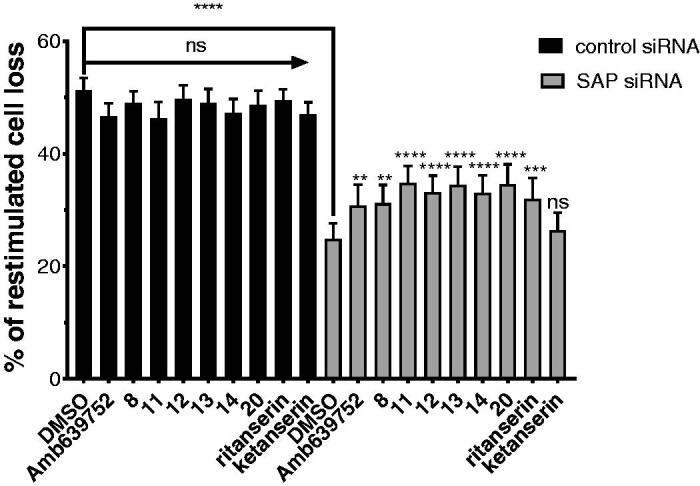
DGKα inhibitors partially restores apoptosis in SAP deficient lymphocytes. Lymphocytes from normal subjects were transfected with control or SAP specific siRNA (■ control siRNA, ■ SAP siRNA). After 4 days, the cells were restimulated with CD3 agonist OKT3 (10 ng/mL) in presence of respective inhibitor. Vehicle (DMSO) was used as control. Twenty-four hours later, the % of cell loss was evaluated by PI staining. Data are the mean ± SEM of nine independent experiments performed in triplicate.

To evaluate inhibitor efficacy in physiological context, we modelled XLP-1 by silencing SAP in primary peripheral blood T lymphocytes (PBLs) and restimulating them with anti-CD3 antibody (OKT3 10 ng/mL, 24 h). We pre-treated the cells with the indicated inhibitors for 30 min at a concentration of 10 µM^8^. In control siRNA-transfected cells, DGKα inhibitors poorly affect RICD, with Amb639752, **11** and **14** slightly reducing it (Figure 9). Conversely, DGKα inhibitors significantly rescued the apoptotic defect of SAP-deficient T cells although not reaching control levels. At 10 µM, all the new molecules showed an efficacy comparable to Amb637952 and ritanserin used as positive reference molecules. Conversely, the serotonin antagonist ketanserin is inactive, excluding the involvement of serotonin receptors in rescuing the RICD in SAP-deficient T cells (Figure 9).

In summary, these data confirm that the newly identified DGKα inhibitors can rescue RICD susceptibility in T cell models of XLP-1 suggesting a putative use for XLP-1 therapy.

### 3.5. DGKα inhibitors reduce migration of the cancer cells (MCF7)

**Figure 10.**
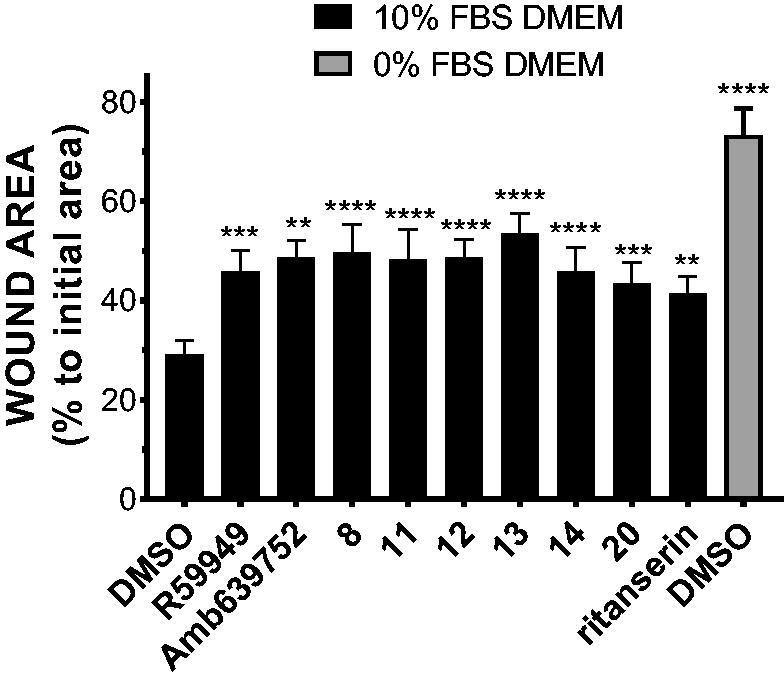
Novel DGKα inhibitors slow tumour cell migration. MCF7 monolayer was wounded and treated for 15 h with serum in presence of our new DGKα inhibitors (10 μM) or vehicle (DMSO). Results are expressed as the percentage of wound area compared to the initial area. Data are the mean ± SEM of nine independent experiments.

Previous studies conducted in our laboratory demonstrated that the inhibition of DGK activity decreases chemotaxis, proliferation, migration, and invasion of many cancer cell lines^12–14^. To evaluate if our newly synthetised DGKα inhibitors were effective in impairing cancer cell migration, we measured serum induced wound healing in MCF7 breast cancer cells in presence of 10 µM inhibitor. In presence of serum, none of the inhibitors is toxic for MCF7 cells even after prolonged treatment (data not shown). After 15 h of treatment, all the newly synthesised active molecules equally reduced cell migration when compared to the vehicle (DMSO) delaying wound closure (Figure 10).

Besides being in good agreement with the notion that DGKα is required for cancer cells migration, this observation indicates that our new DGKα inhibitors reduce cancer cell motility, suggesting a potential utility in a metastasis setting.

### 3.6. Generation of a pharmacophore hypothesis

**Figure 11.**
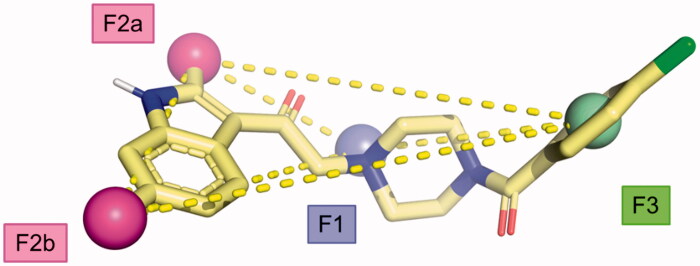
Proposed pharmacophoric model.

From the data obtained, it is possible to identify some key pharmacophoric points crucial for the biological activity of the Amb compounds on DGKs namely: (i) a basic nitrogen; (ii) the methyl groups at the 2 and 6 position of the indole nucleus, and (iii) a (hetero)aromatic ring. This information allows us to build a four-point pharmacophoric model represented in Figure 11 superimposed with the minimised structure of compound **11**. Although, we are not able to evaluate the importance of the two carbonyl groups, it represents the first attempts in order to identify the minimum structural request to interact with DGK catalytic site considering the molecular structure of the four most active inhibitors discovered to date (Amb639752, ritanserin, R59022, and R59949). We feel that this model might be useful to identify novel compounds active on DGKα through more targeted virtual screening campaigns, overcoming the current scaffolds.

## 4. Discussion

As a key component of several signal transduction pathways, DGKα represent an emerging pharmacological target. We have demonstrated the efficacy of DGKα inhibitors for XLP-1 treatment^8^, while others have proposed them for cancer treatment^22^ and to remove immune-checkpoints promoting immune vigilance against cancer^40^. Commercially available DGKα inhibitors are limited by poor specificity^25^^,^^41^ and pharmacokinetic^20^. The CU-3 molecule described by other features a noteworthy activity and specificity but its reactive chemical structure make unlikely an *in vivo* use^30^. With intent of developing molecules suitable for therapeutic use we selected Amb639752 as a novel inhibitor with remarkable DGKα activity. Amb639752 also features improved selectivity for DGKα as it does not affect serotonin signalling^9^. Despite numerous efforts a structure of mammalian DGKs is still missing, thus we decided to explore the structure–activity relationship of this molecule to improve its activity and pave the way for further developments. Our efforts allowed us to build a pharmacophoric model for DGKα inhibitors characterised by three required features. We also characterised a set of novel compounds with improved IC_50_ in the low µM range and identified the most profitable synthetic route for them. The mode of DGKα inhibition by those molecules is still unknown apart for ritanserin, which binds at the same time the DGKα catalytic accessory domain and the C1 domain putatively promoting a close inactive conformation^41^.

The second-generation inhibitors we described in this work maintain the specificity of Amb639752 as they not affect DGKζ, the predominant isoform of lymphocytes^42^ and the broadly expressed DGKθ^43^. Those DGKα inhibitors are active in a lymphocyte based XLP-1 assay and in a cancer cell migration assay, holding the promise for a potential therapeutic application. However, their efficacy is still to be determined in *in vivo* models of disease where some of the parental compounds showed efficacy but poor pharmacokinetic^8^^,^^20^.

## Supplementary Material

Supplemental Material
