# Supplementary material for: Structure activity relationship studies on Amb639752: toward the identification of a common pharmacophoric structure for DGKα inhibitors
Source: J Enzyme Inhib Med Chem. 2019 Nov 5;35(1):96–108. doi: 10.1080/14756366.2019.1684911 (PMC6844378; doi:10.1080/14756366.2019.1684911)
Supplement: Supplemental Material [file IENZ_A_1684911_SM5976.pdf]

SUPPORTING INFORMATION

Table of Contents

p. S2: Synthetic procedures for compounds **1**

p. S6: Synthetic procedures for compounds **2**

Scheme of synthesis for 3-(2-(4-(bis(4-fluorophenyl)methylene)cyclohexyl)ethyl)-2-thioxo-2,3-dihydroquinazolin-4(1H)-one (**1**)

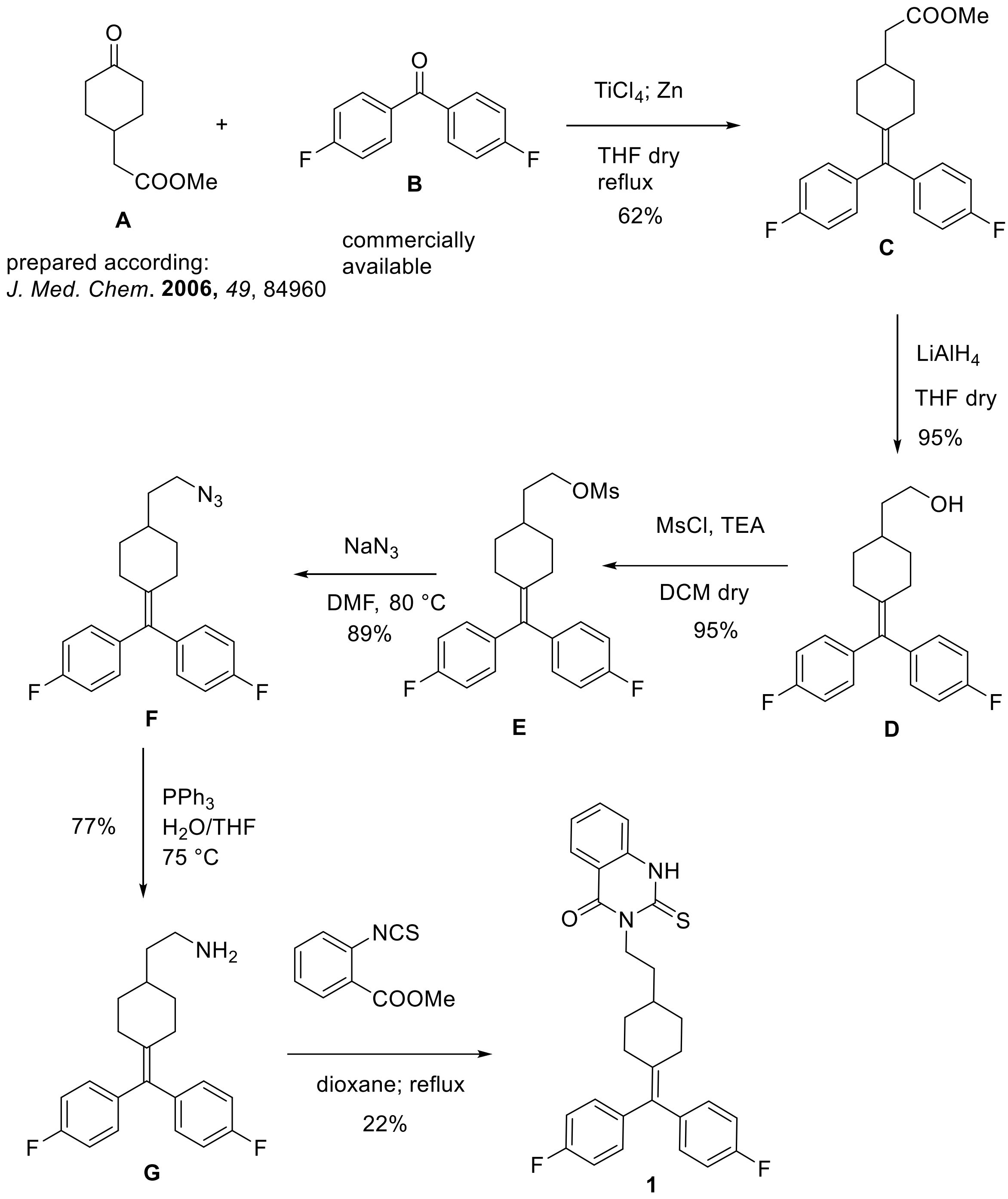

Synthesis of methyl 2-(4-(bis(4-fluorophenyl)methylene)cyclohexyl)acetate (**C**)

Under a nitrogen atmosphere, a THF dry suspension containing 7.7 eq of activated zinc was cooled at -10 °C then 3.7 eq of titanium (IV) chloride were added dropwise. The resultion suspension was

heated at reflux for 2h, than was cooled at room temperature and a solution containing 1 eq of methyl 2-(4-oxocyclohexyl)acetate and 1 eq of bis(4-fluorophenyl)methanone was added dropwise. The reaction was heated at reflux for 3 h. The reaction was then worked up by dilution with diethylether and $NaHCO_3$ sol. sat, filtered on a celite pad. The aqueous phase was washed with diethylether (x3) and the combined organic phases washed with brine (x1) and dried over sodium sulphate. The crude was finally purified by column chromatography using PE/EtOAc 98:2 and 95:5 as eluants to give **C** as white solid.

^{1}H-NMR: (300 MHz, CDCl3) δ 7.26-7.01 (m, 4H), 7.01-6.75 (m, 4H), 3.66 (s, 3H), 2.69-2.42 (m, 2H), 2.42-2.18 (br d, 2H), 2.17-1.70 (m, 5H), 1.36-1.04 (m, 2H).

^{13}C-NMR: (75 MHz, CDCl3) δ 173.4, 161.6 (d, *J* = 243.9 Hz), 131.3 (d, *J* = 7.4 Hz), 138.7, 133.2, 131.3, 115.0 (d, *J* = 21.2), 51.5, 41.2, 34.8, 34.4, 31.4.

IR: (KBr) ν 1735, 1506, 1215, 1081, 842, 558 cm^{-1}

Melting point: 116.1-117.2 °C

Synthesis 2-(4-(bis(4-fluorophenyl)methylene)cyclohexyl)ethan-1-ol (**D**)

Under a nitrogen atmosphere, the corresponding ester (**C**) was dissolved in THF dry (0.3 M). The resulting solution was cooled at 0 °C and 1.1. eq of $LiAlH_4$ were added portionwise. After 2 h at room temperature, the reaction was quenched adding a saturated aqueous solution of Na_2SO_4. The resultion solution was extracted with EtOAc (x3) and the combined organic phases were washed with brine (x1) and dried over sodium sulphate. The crude was purified by column chromatography using PE/EtOAc 8:2 as eluent to give the desired compound as colorless oil.

^{1}H-NMR: (300 MHz, CDCl3) δ 7.19-7.01 (m, 4H), 6.95-6.90 (m, 4H), 3.65 (t, *J* = 6.5 Hz, 2H), 2.52 (br d, 2H), 2.00-1.76 (m, 4H), 1.76-1.58 (m, 1H), 1.49 (q, *J* = 6.7 Hz, 2H), 1.30-1.19 (m, 2H).

^{13}C-NMR: (75 MHz, CDCl3) δ 161.4 (d, *J* = 243.3 Hz), 139.4, 138.7, 132.6, 131.2 (d, *J* = 7.4 Hz), 114.8 (d, *J* = 20.6 Hz), 60.4, 39.4, 34.7, 34.1, 31.5.

IR: ν 3338, 1504, 1218, 1154, 833, 799 cm^{-1}

Synthesis of 2-(4-(bis(4-fluorophenyl)methylene)cyclohexyl)ethyl methanesulfonate (**E**)

1 eq of alcohol **D** was dissolved in dichloromethane dry (0.3 M), the resulting solution was cooled at 0 °C and 2 eq of TEA and 1.5 eq of MsCl were subsequently added. The reaction was stirred for 1 h at room temperature under a nitrogen atmosphere, then was diluted with water and extracted with dichloromethane (x3). The combined organic phases were washed with brine (x1) and dried over sodium sulphate. The crude was purified by column chromatography using PE/EtOAc 8:2 as eluant to give the desired product as yellowish oil.

^{1}H-NMR: (300 MHz, CDCl3) δ 7.14-6.98 (m, 4H), 6.98-6.86 (m, 4H), *4*.24 (t, *J* = 6.0 Hz, 2H), 2.94 (s, 3H), 2.51 (br d, 2H), 2.08-1.76 (m, 4H), 1.80 (br d, 1H), 1.66 (br t, 2H), 1.21-1.03 (m, 2H).

^{13}C-NMR: (75 MHz, CDCl3) δ 161.1 (d, *J* = 243.3 Hz), 138.6, 138.4, 132.6, 131.0 (d, *J* = 7.4 Hz), 114.6 (d, *J* = 21.2 Hz), 68.2, 36.7, 35.3, 33.9, 33.6, 31.0.

IR: ν 2360, 2336, 1504, 1217, 1173, 831, 798 cm^{-1}

Synthesis of 4,4'-((4-(2-azidoethyl)cyclohexylidene)methylene)bis(fluorobenzene) (**F**)

The mesylate **E** was dissolved in DMF (0.4 M) and 1.2 eq of sodium azide were added. The solution was heated at 80 °C overnight. The reaction was then diluted with water and extracted with EtOAc (x3). The combined organic phases were washed with water (x3), brine (x1) and dried over sodium sulphate. The crude obtained as yellowish oil was sufficiently pure to be used in the next step.

^{1}H-NMR: (300 MHz, CDCl3) δ 7.27-7.08 (m, 4H), 7.08-6.95 (m, 4H), 3.33 (br t, 2H), 2.59 (br d, 2H), 2.10-1.91 (m, 2H), 1.90-1.78 (br d, 2H), 1.77-1.51 (m, 3H), 1.18-1.05 (m, 2H).

^{13}C-NMR: (75 MHz, CDCl3) δ 161.5 (d, *J* = 243.3 Hz), 139.0, 138.7, 132.9, 131.3 (d, *J* = 7.4 Hz), 114.9 (d, *J* = 21.2 Hz), 49.2, 35.3, 34.9, 34.3, 31.4.

IR: ν (KBr) 2161, 2120, 1504, 1218, 1154, 836, 559 cm^{-1}

Synthesis 2-(4-(bis(4-fluorophenyl)methylene)cyclohexyl)ethan-1-amine (**G**)

The azide **F** was dissolved in THF (0.3 M). At the resulting solution 1.5 eq of triphenylphosphine and 6 eq of water were added. The reaction was heated at reflux for 2 h. After evaporation of the solvent, the crude was purified by column chromatography using PE/EtOAc 2:8 and EtOAc/MeOH 8.2 as eluants to give the desired amine as white solid.

^{1}H-NMR: (300 MHz, CD3OD) δ 7.12-7.05 (m, 4H), 7.03-6.96 (m, 4H), 2.95 (br t, 2H), 2.54 (br d, 2H), 2.10-1.95 (m, 2H), 1.95-1.80 (m, 2H), 1.75-1.50 (m, 3H), 1.28-1.08 (m, 2H).

^{13}C-NMR: (75 MHz, CD3OD) δ 161.5 (d, *J* = 243.3 Hz), 138.8, 133.1, 131.2 (d, *J* = 7.4 Hz), 114.5 (d, *J* = 21.2 Hz), 37.6, 34.8, 33.9, 31.0.

IR: ν (KBr) 3440, 3195, 1505, 1223, 1155, 835, 557 cm^{-1}

Melting point: 185.5-188.6 °C

Synthesis of 3-(2-(4-(bis(4-fluorophenyl)methylene)cyclohexyl)ethyl)-2-thioxo-2,3-dihydroquinazolin-4(1H)-one (**1**)

The amine **G** was dissolved in dioxane (0.3 M) and 2 eq of methyl 2-isothiocyanatobenzoate were added. The reaction was heated at reflux overnight. After dilution with water, the solution was extracted with EtOAc (x3). The combined organic phases were washed with brine (x1) and dried over sodium sulphate. The crude was purified by column chromatography using PE/EtOAc 95:5 and 9:1 as eluants to give compound **1** as white solid.

^{1}H-NMR: (300 MHz, (CD3)2CO) δ 11.62 (s, 1H, NH), 8.07 (d, *J* = 7.9 Hz, 1H) 7.73 (t, *J* = 7.6 Hz, 1H), 7.44 (d, *J* = 6.0 Hz, 2H), 7.34 (t, *J* = 7.8 Hz, 2H), 7.29-7.13 (m, 6H), 4.53 (t, J = 6.6 Hz, 2H), 2.52 (br d, 2H), 2.20-1.85 (m, 2H), 1.81-1.51 (m, 3H), 1.31-1.10 (m, 4H).

IR: (KBr) ν 3174, 1688, 1504, 1224, 1143, 828, 758 cm^{-1}

Melting point: 215.9-219.1 °C

MS (ESI) *m/z* 489 $(M+H)^+$

Synthetic scheme for 2-(4-(furan-2-carbonyl)piperazin-1-yl)-1-(1H-indol-3-yl)ethan-1-one (**2**)

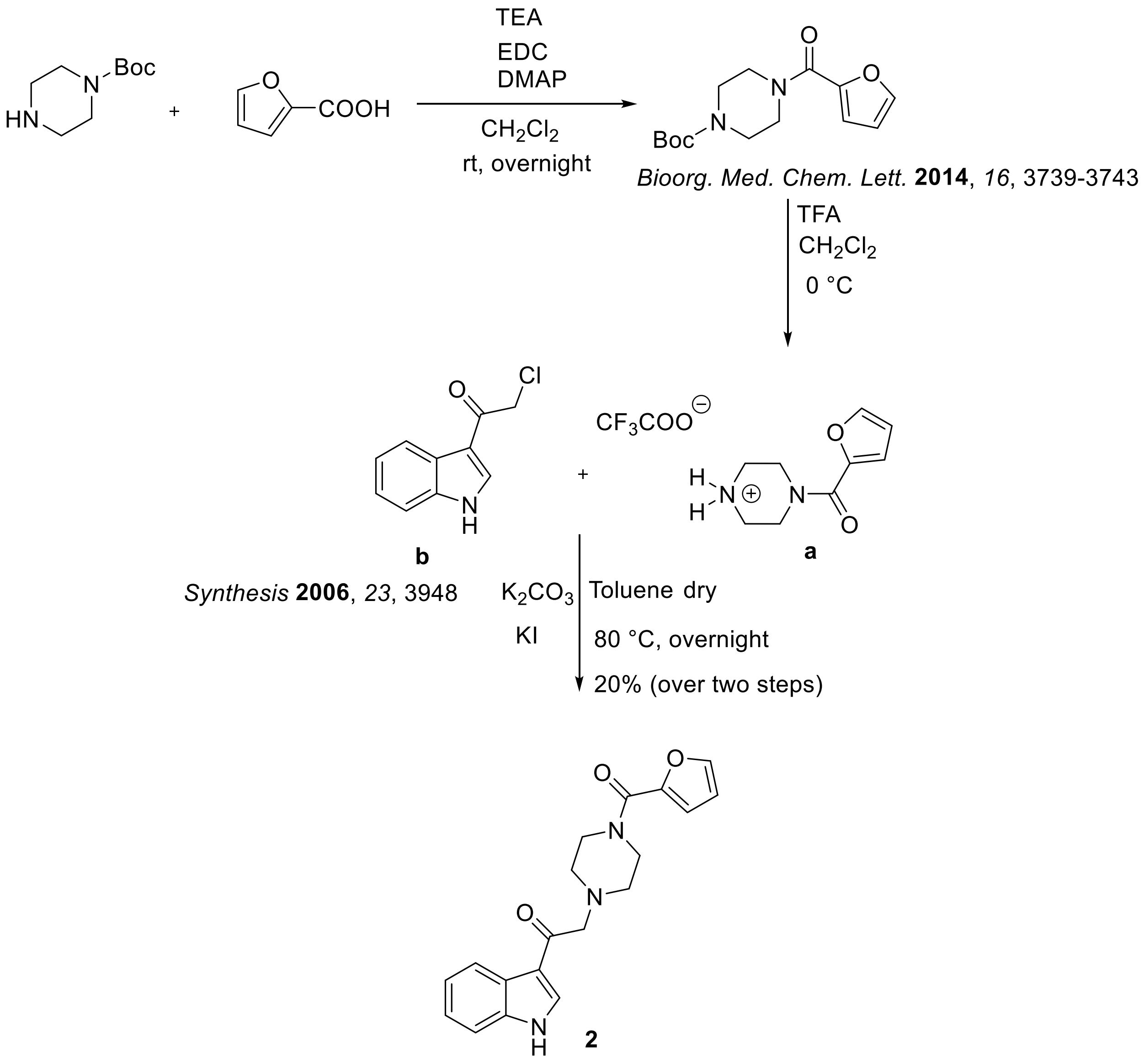

Synthesis of 4-(furan-2-carbonyl)piperazin-1-ium trifluoroacetate (**a**)

The Boc-piperazine derivative was dissolved in dichloromethane dry (0.3 M). To the resulting solution, cooled at 0 °C, 16 eq of trifluoroacetic acid were added. The reaction was stirred for 3 h at 0 °C. Evaporation of the solvent, gave a solid, which was used as such for the following step.

Synthesis of 2-(4-(furan-2-carbonyl)piperazin-1-yl)-1-(1H-indol-3-yl)ethan-1-one (**2**)

1 eq of compound **b** and 1.5 eq of compound **a** were dissolved in toluene dry (0.3 M) then 1 eq of potassium carbonate, 2.5 eq of potassium iodide were added. The reaction was heated at 80 °C overnight. Evaporation of the solvent gave a crude which was directly purified by column chromatography using PE/EtOAc 1:9 and EtOAc as eluants to give **2** as yellowish solid in 20% yield over two steps.

^{1}H-NMR: (300 MHz, $CDCl_3$) 9.76 (br s, NH), 8.37 (br d, 1H), 8.13 (s, 1H), 7.46 (s, 1H), 7.40 (m, 1H), 7.26 (m. 2H), 6.98 (br d, 1H), 6.46 (br d, 1H), 3.85 (br s, 4H), 3.70 s, 2H), 2.67 (br s, 4H)

IR: (KBr) ν 3185, 1629, 1420, 1308, 1113, 938, 747 cm^{-1}

Melting point: 158.2-159.1 °C

MS (ESI) *m/z* 338 $(M+H)^+$

Second synthetic route for the synthesis of **8**

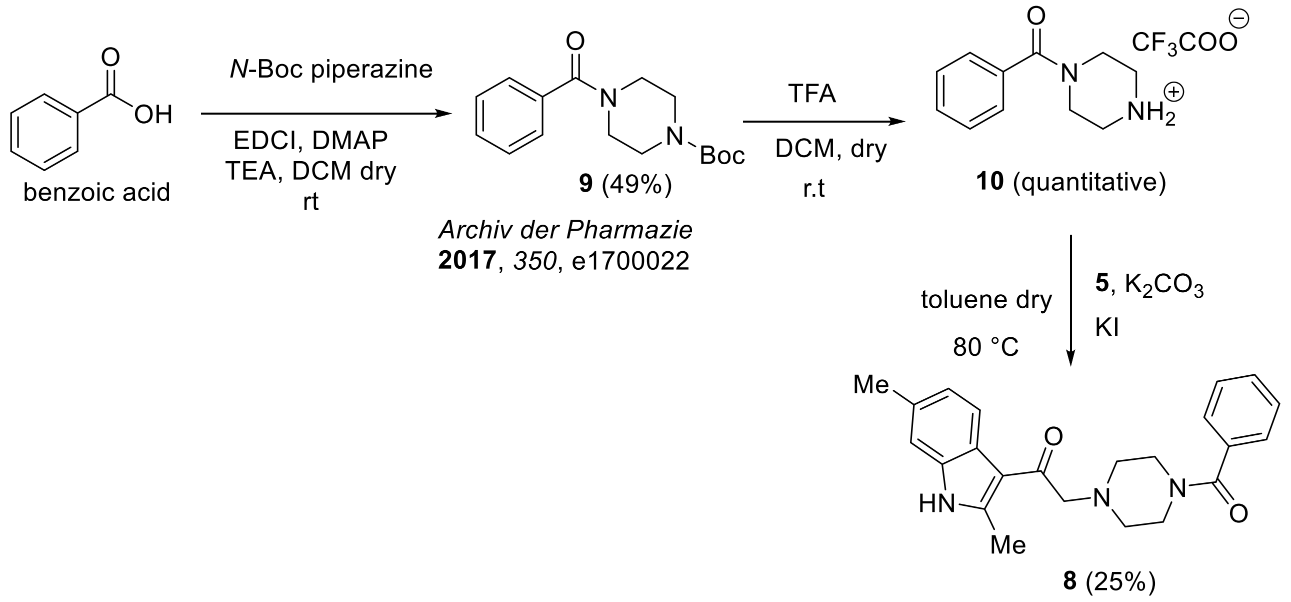

Synthesis of 2-(4-benzoylpiperazin-1-yl)-1-(2,6-dimethyl-1H-indol-3-yl)ethan-1-one (***8***)

1 eq of compound **10** and 1 eq of compound **5** were dissolved in toluene dry (0.3 M) then 2.5 eq of potassium carbonate, 0.1 eq of potassium iodide were added. The reaction was heated at 80 °C

overnight. Evaporation of the solvent gave a crude which was directly purified twice by column chromatography using EtOAc and EtOAc/MeOH 9:1 as eluants to give **8** as white solid in 25% yield.
